# Characteristics and Demographics of Patients Requiring Emergent Air Medical

**DOI:** 10.1155/2022/3044891

**Published:** 2022-03-01

**Authors:** Anna Van Tuyl, Mark Quilon, Trevor Dudley, Olivia Grant, Neha Rao, Paul Barbara, David S. Kugler, Kaitlin C. McLoone-Cepin, Josh Greenstein, Barry Hahn

**Affiliations:** ^1^Department of Emergency Medicine, Staten Island University Hospital, Northwell Health, Staten Island, New York, NY, USA; ^2^Department of Emergency Medicine, Plainview Hospital, Northwell Health, Plainview, New York, NY, USA; ^3^Department of Helicopter Emergency Medical Services, Northwell Health, Syosset, New York, NY, USA

## Abstract

**Background:**

As integrated health systems become more common, interfacility patient transfers will increase and air transport programs will be prioritized. Understanding characteristics of patients triaged to air medical transport will assist with resource allocation and needs assessment. The objective of this study was to investigate the demographics and clinical characteristics of patients that presented to the emergency department (ED) and subsequently required emergent air medical interfacility transport.

**Methods:**

This was a retrospective, multicenter study conducted at eight hospitals within Northwell Health, the largest academic health system in New York state. The study was conducted between December 1, 2014, and July 31, 2020, and included patients who presented to an ED and subsequently required emergent air medical interfacility transport.

**Results:**

Overall, the median age was 37 years (IQR 4–66), and 231 (54%) subjects were males. The majority of subjects (59%) had no reported comorbidities, arrived by ambulance (52%), and were emergency severity index triage 2 (48%). Frequent indications for transfer were nontraumatic neurologic (37%), pulmonary or respiratory (13%), trauma (12%), and cardiovascular (12%). Most patients were not ventilated before transport (71%). The median time to call for transport at the sending institution was 2:42 hours (IQR 1:14–6:54), and the median length of stay was 4:12 (IQR 2:31–8:48). Most patients were subsequently admitted (96%) at the receiving institution to an intensive care unit (72%).

**Conclusions:**

This study describes patients' demographic and clinical characteristics who required emergent air medical transport. Helicopter transport is costly, and data from these patients may further help our understanding of who is transported by air and how important air transport is to the health system.

## 1. Introduction

Since the 1960s, rotor-wing aircraft has been used to transport patients in the prehospital setting [1]. Air medical transport is now a well-established part of the emergency medical services (EMS) system and has evolved to include interfacility transfers. Large urban areas may not utilize aircraft as often as other systems due to short scene-to-hospital ground transport times and the density of multiple local hospital facilities [2]. However, sharing resources and services in a large health system depends on transporting patients to partner hospital facilities. As large, integrated health systems become more common, the need for patient transfers between facilities will increase, and the development of air transport programs will be prioritized. Regionalization of care and the requirement for specialized resources results in the frequent need for interfacility transport of critically ill patients [3]. Patients at more remote hospitals within the health system may require transfer to tertiary care centers or trauma centers for specialized care.

Previous literature has demonstrated that hospitals in large urban settings may not utilize aircraft transport as often as other systems. In these environments, the total time necessary to transport patients via air may be more time-consuming than transport by ground. This is thought to be due to the density of hospitals, short ground ambulance times in urban areas, and air medical transport requires multiple patient transfers involving remote helipads and transport of ambulance needed [2]. Additionally, in an analysis of air and ground transfer of nonpenetrating trauma patients, air transport has led to longer total transport times in facilities without on-site helipads [4]. One study of interfacility helicopter transport for neurosurgical patients to a single trauma center found 63% of patients could have been safely stabilized at the referring hospital or undergone ground transfer. The median times to neurosurgical intervention at the accepting hospital were long enough. Even if air transport led to modest reductions in transfer time, this did not benefit most patients [5]. A substantial number of factors need to be considered when evaluating a patient for interfacility air transport.

There is a lack of literature on utilizing interfacility transfers from the emergency department (ED) to tertiary care centers. Understanding characteristics of patients triaged to air medical transport will assist with appropriate resource allocation and need assessment. Interfacility helicopter transfer of patients can play a critical role in delivering specialized care. However, it does not offer a substantial benefit for some, and the patients can be safely and expeditiously transported by other means. This information can help develop transport decision-making processes to optimize transport mode appropriateness and maximize resource utilization. The Northwell Health system, which comprises urban, suburban, and rural hospitals in and around the New York area, utilizes an air transport system for transfers between healthcare facilities as a part of their EMS system. The primary objective of this study was to investigate the demographics and clinical characteristics of patients that presented to the ED and subsequently required emergent air medical interfacility transport.

## 2. Materials and Methods

### 2.1. Study Design and Setting

This was a retrospective, multicenter study conducted at eight hospitals within Northwell Health, the largest academic health system in New York state. The study was conducted between December 1, 2014, and July 31, 2020, and included patients who presented to an ED and subsequently required emergent air medical interfacility transport. This period was identified because the current electronic health record (EHR), Allscripts Sunrise (Allscripts, Chicago, IL), was utilized by most hospitals within the system during this period. Transfer from or to hospitals outside the Northwell Health system was not included in the database to avoid incomplete records. Hospitals within the Northwell Health system that did not use the current EHR within this period were also not included. Excluding these institutions allowed for a comprehensive electronic database. The local institutional review board approved this study.

### 2.2. Selection of Participants

All subjects who presented to the ED and subsequently required emergent air medical interfacility transport were included in the study. Charts with incomplete data were excluded from this study. If it was determined that the same subject was evaluated in the ED and transported for a new incident, they were permitted to be included again in the study.

### 2.3. Measurements

Four study members trained in the study protocol and data abstraction each reviewed patient records. We utilized a predesigned, standardized case report form to record the data from the electronic chart reports. An additional senior investigator checked data input accuracy for 10% of all patients to eliminate errors and ensure consistency and accuracy. Any discrepancies were adjudicated by reviewing the patient's medical chart.

Patient demographics that were collected included were age, gender, ethnicity, date of service, insurance status, comorbidities, time of onset, mechanism of injury or illness, mode of transport, length of stay (LOS), and treatment before and after transport. Procedures or interventions identified but not listed individually included cardioversion, sedation, joint reduction, cardiac pacing, nasogastric tube, chest tube placement, central venous catheter, and electrocardiograms. The emergency severity index (ESI) was also extracted from charts. ESI is a five-level emergency department triage algorithm based on the acuity of a patient's healthcare problems, and the number of resources their care is anticipated to require. The ESI levels are numbered one through five, with level one indicating the greatest urgency [6]. All results were reported as composite data and also stratified by age, gender, and ethnicity.

### 2.4. Data Collection and Processing

The data were collected and managed using Research Electronic Data Capture (REDCap), a secure, web-based application designed to support data capture for research studies.

The data were analyzed using descriptive statistical methods and were expressed as frequency counts and percentages for categorical variables or as mean and standard deviation or median and interquartile range (IQR), as appropriate, for continuous variables. Data analyses were conducted using the Analyse-it version 4.95.4 (Analyse-it Software, Leeds, UK).

## 3. Results

During the study period, 2441 patients presented to the ED and subsequently required emergent air medical interfacility transport. Of these, 1504 were excluded since they were no interfacility transports within the Northwell Health system, leaving 937 Northwell Health interfacility transports. Other 511 charts were incomplete, leaving 426 charts for inclusion in the final review.

### 3.1. Demographic Characteristics

Overall, the median age was 37 years (IQR 4–66), and 231 (54%) subjects were males. The majority of subjects (59%) had no reported comorbidities, arrived by ambulance (52%), and were ESI 2 (48%). Among pediatric patients aged 0–21 years, the median age was two years (IQR 0.1–9), and 107 (60%) subjects were males. Among adults, the median age was 62 years (IQR 49–73), and 124 (50%) subjects were males. The adult cohort tended to have cardiac comorbidities (49%). Arrival via ambulance was the most common mode of arrival to the hospital in the composite group (52%) and all subgroups except the Hispanic and Asian groups. A personal auto was the most common mode of arrival to the hospital for the Hispanic (50%) and Asian groups (50%). In contrast to the other groups, the Asian group also had a lower incidence of level 2 designations (20% versus 48%) and a higher incidence of level 3 designations (30% versus 15%). The remaining demographic characteristics among all groups appeared similar. Complete demographic data for these subjects are given in Table 1.

### 3.2. Clinical Characteristics

Among all subjects, the median systolic blood pressure was 128 (IQR 106–149), and the median heart rate was 102 (IQR 81–135). The most common indications for transfer were nontraumatic neurologic (37%), pulmonary or respiratory (13%), trauma (12%), and cardiovascular (12%). Most patients were not ventilated before transport (71%). The median time to call for transport at the sending institution was 2:42 hours (IQR 1.14–6.54), and the median length of stay was 4:12 (IQR 2.31–8.48). Most patients were subsequently admitted (96%) at the receiving institution to an intensive care unit (72%).

Except for the pediatric and two ethnicity groups, all subgroups had similar incidences of treatments and procedures performed before transport. The pediatric group was less likely to undergo radiographic studies (83%) or individual procedures (45%) compared to the composite group (91% and 48%, respectively). Compared to the incidence of procedures and interventions performed in the composite group (48%), Black patients had a lower incidence (36%), while Asian patients had a higher incidence (80%). The remaining clinical characteristics among all groups appeared similar. Complete clinical data for these subjects are given in Table 2.

## 4. Discussion

This study aimed to investigate patients' demographics and clinical characteristics that presented to the ED and subsequently required emergent air medical interfacility transport. This study looked at 426 patients transported by helicopter between facilities within the Northwell Health system from 2014 to 2020. Northwell Health is situated in the urban and suburban parts of New York City and surrounding areas. Due to traffic and city roads, ground transportation is hindered by long travel times. When the decision to transport a patient to a higher level of care at a different facility is made, air transport is often chosen due to its quick transport time. This decision to utilize air transport can be essential when the patient requires a procedure or intervention limited by time or if the patient's condition is at high risk of deterioration during transport.

Amongst other studies, this study is unique in looking at interfacility air transport within a large healthcare system with a sizable group of pediatric patients. Similar studies only look at transport between hospitals from different health systems. In addition to characteristics looked at by other studies, the current study gathered information about the race and ethnicity of the patient population.

The study found the median age of transported patients to be 37, with 54% male compared to similar studies. This study skews much younger than other studies with a more even distribution of male and female patients. Di Rocco et al. looked at a population with a mean age of 51, and 61% were male [7]. Alstrup et al. report two separate studies where the population had a median age of 60 and males composed 71% in one study and 64% in the second study [8, 9]. The pediatric patient population had a median age of two in this study, with 60% male participants. On the other hand, Alink et al. looked at a pediatric patient population with a mean age of six, 59% male [10].

Most patients in the present study were triaged at level 2, around the middle of the acuity levels. However, transported patients were subsequently determined to be acutely ill, with over 90% admitted to the hospital and over 70% admitted to an intensive care unit (ICU) level of care. Alstrup et al. describe similar findings in a study of several medical centers in Denmark, where 85% of airlifted patients were hospitalized at university hospitals, and 31% were admitted to an ICU level of care. Also, in the study by Alstrup et al., most patients airlifted did not require mechanical ventilation, in agreement with our research, 71% were not ventilated [9].

In the pediatric population, most were found to have no comorbidities (85%). However, in adults, subjects most commonly had cardiac comorbidities (49%). Pediatric patients were mainly transported for respiratory issues (22%) and pediatric and neonatal issues (22%). Adult patients were mainly transported for nontraumatic neurologic and cardiac problems, respectively, at 54% and 16%. These findings correlate with other studies. Alink et al. found that among pediatric patients using helicopter transport, 11% were pediatric resuscitations and 18% were combined airway and respiratory problems [11]. Alstrup et al. found that of adult patients using air transport, 27% had a myocardial infarction (MI), 17% had a cardiac arrest, and 21% had a stroke [9]. However, Alink et al. found that trauma accounted for 54% of diseases that required helicopter transport in pediatric patients [11]. In this study, only 14% of pediatric patients were sent by air transport for trauma.

The flight duration was not gathered in this study, but the time to call for transport and length of stay at the sending facility was long. It took a median time of 2 hours and 42 minutes to call for transport at the sending facility. The patient spent a median duration of 4 hours and 12 minutes before helicopter transport came. This duration may be excessive for time-critical procedural rescue situations such as interventional vascular work not available at a sending facility, such as percutaneous interventions management of acute ischemic conditions, acute surgical services, or other specialist-based interventions of life-threatening conditions. However, for other time-sensitive (but not critical) high-risk patient care transfers, this time interval may represent the acquisition of special personnel or resources to handle the patient's needs. Examples include transfers utilizing individual resources such as ventricular assist devices, balloon pumps, unique ventilator setups, and extracorporeal membrane oxygenation systems. The helicopter cannot be reasonably equipped with all of these devices for every activation. In addition to limited resources being deployed to a remote patient for safe transfer, specific patient subsets require additional staff as per regional policies, including but not limited to pediatric cases (such as neonatal intensive care). It is a distinct benefit of a helicopter transfer service to reduce the patient's riskiest time interval, the time spent out of the hospital, even if the total transfer time from call to arrival is equivalent to ground services. Our study did not parse out the indication for transfer nor the sending clinician's expectations on time to complete transfer which could further categorize our result. Hence, the result is helpful for descriptive understanding. Quality programs at a similar service should identify time interval goals that are most relevant to optimal patient care vs. applying time statistics to every transfer to understand that immediate arrival is not always the correct deployment for the patient's needs.

For interfacility transports from slightly more distant sites, it is commonly assumed that helicopter transport is faster than traditional transport. Out-of-hospital time can be considerably shortened because helicopters travel at higher rates of speed, by more direct routes than ground ambulances, and avoid traffic and other road conditions that can slow transport. However, helicopter transport is not always faster. The additional time necessary for initiation of a flight and flight time from the helicopter base to the referring hospital may offset the speed of helicopter flight [11].

With regards to race and ethnicity, this study had several findings. The Hispanic and Asian groups were more likely to arrive at the hospital by personal auto (50%) than the group as a whole, where most arrived by ambulance (52%). The Asian group had higher triage level 3 designations (30%) than level 2 (20%), the opposite of the overall study population, where level 3 designations (15%) were lower than level 2 designations (48%). In addition, the Black patient population had a lower incidence of procedures and interventions (36%) than the study population (48%), whereas the Asian patients had a higher incidence (80%). These findings are novel since few studies look at differences based on race and ethnicity regarding helicopter transport. However, the current results are constrained by the low number of Hispanic and Asian patients in the study population, making up only 4% and 2%, respectively.

There are limited studies on interfacility transfers by helicopter, and most are geared towards a specific disease, such as trauma, stroke, or MI. This study examines a general view of patients transported by helicopter, while other studies, such as by Di Rocco et al., have limited patient demographics. Di Rocco et al. gathered information on flight distances and flight interventions, which this study did not [8]. This study only looked at the time to call for transport at the sending facility and the patient's length of stay at the sending facility before transport arrives. In addition, another study by Ryb et al. gathered data on the survival of patients transported by helicopter, which was not looked at in this study [12]. This type of data may be more clinically relevant.

### 4.1. Limitations

The study has several limitations. First, the study is a retrospective, observational study of a single healthcare system in the northeast United States region. Therefore, the study is limited due to its' retrospective nature. Furthermore, this study may not generalize to other health systems as Northwell Health skews to populations in urban and suburban areas in the New York City area. Next, only 426 charts were reviewed, with many patients excluded due to limited or missing data.

Moreover, this study did not analyze cost and patient safety indicators, which are essential in determining the clinical effectiveness of helicopter transport. In addition, there is a low representation of Hispanic and Asian patients, making up only 4% and 2% of the patient population, respectively. Specific findings, such as the high rate of interventions in Asian patients, regarding these populations are at increased risk for bias because of their small sample sizes. Also, a significant number of the patient population (25%) had no ethnicity given.

## 5. Conclusion

In summary, this study describes patients' demographic and clinical characteristics who presented to the ED and subsequently required emergent air medical interfacility transport within a large healthcare system in Northwell Health and surrounding areas. To improve the effectiveness of interfacility helicopter transfers and maximize resource utilization, understanding and quantifying the characteristics of these patients is necessary. Helicopter transport is costly, and data from these patients may further help our understanding of who is transported by air and how important air transport is to the health system. This data will help form the foundation for future research regarding helicopter transport for interfacility transfers within healthcare systems.

## Figures and Tables

**Table 1:** Demographics characteristics of subjects.

	Composite (*n* = 426)		Pediatrics (0–21) (*n* = 179)		Adults (>21) (*n* = 247)		Males (*n* = 231)		Females (*n* = 195)		White (*n* = 215)		Black (*n* = 67)		Hispanic (*n* = 18)		Asian (*n* = 10)	
Age (median (IQR))	37	(IQR 4–66)	2	(IQR 0.1–9)	62	(IQR 49–73)	36	(IQR 3–62)	40	(IQR 7–71)	56	(IQR 12–71)	18	(IQR 4–60)	16	(IQR 1–32)	35	(IQR 7–70)
Gender																		
Male	231	54%	107	60%	124	50%					118	55%	32	48%	11	61%	5	50%
Female	195	46%	72	40%	123	50%					97	45%	35	52%	7	39%	5	50%

Ethnicity (*n*, %)																		
White	215	50%	68	38%	147	60%	118	51%	97	50%								
Black	67	16%	34	19%	33	13%	32	14%	35	18%								
Hispanic	18	4%	11	6%	7	3%	11	5%	7	4%								
Asian	10	2%	4	2%	6	2%	5	2%	5	3%								
Others/multiracial	9	2%	3	2%	6	2%	3	1%	6	3%								
N/A	107	25%	59	33%	48	19%	62	27%	45	23%								

Insurance status (*n*, %)																		
Medicare/Medicaid	211	50%	82	46%	129	52%	107	46%	104	53%	94	44%	38	57%	7	39%	7	70%
Private	140	33%	69	39%	71	29%	82	35%	58	30%	89	41%	17	25%	6	33%	3	30%
None	26	6%	12	7%	14	6%	14	6%	12	6%	9	4%	3	4%	2	11%	0	0%
N/A	49	12%	16	9%	33	13%	28	12%	21	11%	23	11%	9	13%	3	17%	0	0%

Comorbidities (*n*, %)																		
None	253	59%	152	85%	101	41%	141	61%	112	57%	119	55%	34	51%	14	78%	5	50%
Cardiac	130	31%	9	5%	121	49%	68	29%	62	32%	77	36%	23	34%	3	17%	3	30%
Diabetes	46	11%	3	2%	43	17%	24	10%	22	11%	18	8%	10	15%	2	11%	3	30%
Pulmonary disease	44	10%	16	9%	28	11%	24	10%	20	10%	18	8%	18	27%	2	11%	1	10%
Renal disease	16	4%	0	0%	16	6%	10	4%	6	3%	11	5%	2	3%	0	0%	0	0%
Coagulopathy	8	2%	1	1%	7	3%	2	1%	6	3%	6	3%	0	0%	0	0%	0	0%
Liver disease	6	1%	2	1%	4	2%	5	2%	1	1%	4	2%	1	1%	0	0%	0	0%

Prehospital mode of transport (*n*, %)																		
Ambulance	220	52%	54	30%	166	67%	112	48%	108	55%	119	55%	37	55%	5	28%	4	40%
Personal auto	84	20%	47	26%	37	15%	47	20%	37	19%	35	16%	13	19%	9	50%	5	50%
Taxi	2	0.5%	0	0%	2	1%	1	0.4%	1	1%	1	0.5%	1	1%	0	0%	0	0%
Bus	1	0.2%	0	0%	1	0.4%	0	0%	1	1%	1	0.5%	0	0%	0	0%	0	0%
N/A	119	28%	78	44%	41	17%	71	31%	48	25%	59	27%	16	24%	4	22%	1	10%

Triage level (*n*, %)																		
4	11	3%	8	4%	3	1%	5	2%	6	3%	5	2%	2	3%	0	0%	1	10%
3	65	15%	35	20%	30	12%	34	15%	31	16%	24	11%	14	21%	5	28%	3	30%
2	204	48%	62	35%	142	57%	112	48%	92	47%	112	52%	32	48%	7	39%	2	20%
1	62	15%	23	13%	39	16%	32	14%	30	15%	33	15%	10	15%	4	22%	2	20%
N/A	84	20%	51	28%	33	13%	48	21%	36	18%	41	19%	9	13%	2	11%	2	20%

**Table 2:** Clinical characteristics of subjects.

	Composite (*n* = 426)	Pediatrics (0–21) (*n* = 179)	Adults (>21) (*n* = 247)	Males (*n* = 231)	Females (*n* = 195)	White (*n* = 215)	Black (*n* = 67)	Hispanic (*n* = 18)	Asian (*n* = 10)
Vital signs (median (IQR))									
Triage BP sys	128 (IQR 106–149)	110 (IQR 86–124)	142 (IQR 123–160)	126 (IQR 102–146)	132 (IQR 111–154)	134 (IQR 109–152)	127 (IQR 112–151)	125 (IQR 118–140)	138 (IQR 123–139)
Triage BP HR	102 (IQR 81–135)	138 (IQR 110–156)	88 (IQR 73–104)	104 (IQR 86–136)	99 (IQR 77–131)	98 (IQR 78–120)	101 (IQR 86–137)	119 (IQR 78–155)	89 (IQR 74–104)

Treatment prior to transport (*n*, %)									
Laboratory studies	418.98	175.98	243.98	224.97	194.99	211.98	66.99	17.94	10.100
Radiographic studies	388.91	149.83	239.97	214.93	174.89	201.93	58.87	15.83	9.90
Medications and transfusions	386.91	163.91	223.90	209.90	177.91	191.89	62.93	18.100	10.100
Procedures and interventions	204.48	80.45	124.50	105.45	99.51	110.51	24.36	8.44	8.80
None	119.28	75.42	44.18	57.25	62.32	49.23	25.37	5.28	0.0
N/A	20.5	1.1	10.4	2.1	0.0	10.5	0.0	0.0	0.0

Indication for transfer (*n*, %)									
Nontrauma neuro	159.37	26.15	133.54	85.37	74.38	87.40	25.37	7.39	2.20
Pulmonary/respiratory trauma	54.13	40.22	14.6	36.16	18.9	22.10	11.16	2.11	1.10
Cardiovascular	51.12	25.14	26.11	29.13	22.11	29.13	4.6	2.11	2.20
Pediatrics/neonatal	49.12	9.5	40.16	34.15	15.8	30.14	6.9	1.6	3.30
GI	39.9	39.22	0.0	17.7	22.11	15.7	6.9	3.17	1.10
OB/GYN	15.4	9.5	6.2	6.3	9.5	5.2	3.4	1.6	0.0
Infections/ID/sepsis	12.3	0.0	12.5	0.0	12.6	5.2	2.3	0.0	0.0
Nontrauma	12.3	10.6	2.1	8.3	4.2	4.2	2.3	2.11	0.0
Surgical	11.3	2.1	9.4	3.1	8.4	8.4	0.0	0.0	1.10
Poisoning/toxic/environmental	11.3	9.5	2.1	5.2	6.3	5.2	3.4	0.0	0.0
EENT	5.1	4.2	10.4	2.1	3.2	2.1	3.4	0.0	0.0
Others	4.1	2.1	2.1	3.1	1.1	2.1	2.3	0.0	0.0
Medical endocrine/diabetic	3.1	3.2	0.0	2.1	1.1	10.5	0.0	0.0	0.0
GU	10.2	1.1	0.0	10.4	0.0	0.0	0.0	0.0	0.0
Metabolic	0.0	0.0	0.0	0.0	0.0	0.0	0.0	0.0	0.0

Ventilated (*n*, %)									
No	303.71	126.70	177.72	164.71	139.71	151.70	51.76	14.78	6.60
Yes	123.29	53.30	70.28	67.29	56.29	64.30	16.24	4.22	4.40

Transport times (median (IQR))									
Triage to call for transport	02:42 (IQR 1:14–6:54)	03:01 (IQR 1:31–8:05)	02:25 (IQR 1:09–6:11)	02:43 (IQR 1:19–6:45)	02:39 (IQR 1:12–6:55)	02:27 (IQR 1:08–8:09)	03:02 (IQR 1:14–7:02)	02:54 (IQR 2:07–4:54)	02:45 (IQR 2:31–3:28)
Length of stay at sending	04:12 (IQR 2:31–8:48)	04:50 (IQR 3:22–9:50)	03:35 (IQR 2:14–7:45)	04:14 (IQR 2:41–8:46)	04:07 (IQR 2:27–8:36)	03:49 (IQR 2:21–9:47)	04:44 (IQR 2:37–8:13)	03:54 (IQR 3:32–6:25)	04:19 (IQR 3:55–5:28)
Call for transport to depart	01:24 (IQR 1:01–1:49)	01:46 (IQR 1:28–2:16)	01:05 (IQR 0:53–1:25)	01:26 (IQR 1:04–1:54)	01:20 (IQR 0:58–1:46)	01:19 (IQR 0:59–1:45)	01:23 (IQR 1:08–1:39)	01:30 (IQR 0:52–1:49)	01:21 (IQR 1:05–2:00)
Transport turnaround time	00:45 (IQR 0:33–1:03)	00:52 (IQR 0:40–1:16)	00:40 (IQR 0:30–0:57)	00:48 (IQR 0:35–1:07)	00:42 (IQR 0:33–1:00)	00:44 (IQR 0:35–1:04)	00:42 (IQR 0:33–0:56)	00:39 (IQR 0:29–1:03)	00:45 (IQR 0:38–1:20)
Transfer time	00:12 (IQR 0:11–0:14)	00:12 (IQR 0:11–0:14)	00:12 (IQR 0:10–0:14)	00:12 (IQR 0:11–0:14)	00:12 (IQR 0:10–0:14)	00:12 (IQR 0:10–0:14)	00:12 (IQR 0:11–0:13)	00:13 (IQR 0:12–0:14)	00:11 (IQR 0:08–0:12)

Disposition (*n*, %)									
Admit	410.96	170.95	240.97	223.97	187.96	208.97	64.96	17.94	9.90
Discharged	11.3	7.4	4.2	5.2	6.3	3.1	2.3	1.6	1.10
Expired	4.1	1.1	3.1	2.1	2.1	3.1	1.1	0.0	0.0
Transfer	10.2	1.1	0.0	10.4	0.0	10.5	0.0	0.0	0.0

Admission location (*n*, %)									
ICU	305.72	131.73	174.70	168.73	137.70	146.68	49.73	13.72	9.90
Med/surg floor	57.13	35.20	22.9	31.13	26.13	31.14	9.13	3.17	0.0
Telemetry	23.5	3.2	20.8	13.6	10.5	16.7	3.4	0.0	0.0
Others	27.6	2.1	25.10	12.5	15.8	17.8	4.6	0.0	0.0
N/A	14.3	8.4	6.2	7.3	7.4	5.2	2.3	2.11	1.10

## Data Availability

The data used to support the findings of this study are available from the corresponding author upon request.

## References

[1] Carter G., Couch R., O’Brien D. J. (1988). The evolution of air transport systems: a pictorial review. *Journal of Emergency Medicine*.

[2] Asaeda G., Cherson A., Giordano L., Kusick M. (2001). Utilization of a Ir Medical Transport in a Large Urban Environment: a Retrospective a nalysis. *Prehospital Emergency Care*.

[3] Blackwell T. H. (2002). Interfacility transports. *Seminars in Respiratory and Critical Care Medicine*.

[4] Hawilo H., Taneja R. (2020). Interfacility helicopter transfers for critically ill patients: always the right choice?. *Critical Care*.

[5] Walcott B. P., Coumans J.-V., Mian M. K., Nahed B. V., Kahle K. T. (2011). Interfacility helicopter ambulance transport of neurosurgical patients: observations, utilization, and outcomes from a quaternary level care hospital. *PLoS One*.

[6] Gilboy N., Tanabe P. https://www.ahrq.gov/professionals/systems/hospital/esi/index.html.

[7] Di Rocco D., Pasquier M., Albrecht E., Carron P.-N., Dami F. (2018). HEMS inter-facility transfer: a case-mix analysis. *BMC Emergency Medicine*.

[8] Alstrup K., Petersen J. A. K., Sollid S., Johnsen S. P., Rognås L. (2020). Mortality and hospitalisation in the Danish Helicopter Emergency Medical Service (HEMS) population from 2014 to 2018: a national population-based study of HEMS triage. *BMJ Open*.

[9] Alstrup K., Rognås L., Sollid S., Johnsen S. P., Valentin J. B., Petersen J. A. K. (2021). Association of helicopter vs ground emergency medical transportation with 1-year mortality in Denmark. *JAMA Network Open*.

[10] Oude Alink M. B., Moors X. R. J., Karrar S. (2021). Characteristics, management and outcome of pre-hospital pediatric emergencies by a Dutch HEMS. *European Journal of Trauma and Emergency Surgery*.

[11] Svenson J. E., O’Connor J. E., Lindsay M. B. (2006). Is air transport faster? A comparison of air versus ground transport times for interfacility transfers in a regional referral system. *Air Medical Journal*.

[12] Ryb G. E., Dischinger P., Cooper C., Kufera J. A. (2013). Does helicopter transport improve outcomes independently of emergency medical system time?. *Journal of Trauma and Acute Care Surgery*.

